# The Launch of *Sarcoma*

**DOI:** 10.1080/13577149778416

**Published:** 1997-03

**Authors:** Martin Robinson

**Affiliations:** YCRC Department of Clinical Oncology Weston Park Hospital NHS Trust Whitham Road Sheffield S10 2SJ UK


*Sarcoma* (1997) **1**, 3

EDITORIAL

### The launch of *Sarcoma*

MARTIN ROBINSON

*YCRC Department of Clinical Oncology, Weston Park Hospital NHS Trust, Sheffield, UK*

The members of the Editorial Board and I welcome you to the first issue of *Sarcoma.* This is a specialist journal that I hope will meet the needs of all who are interested in this group of diseases. The aim of this journal is to provide a focus and forum for all involved in sarcoma research. This includes scientists and clinicians and stretches from molecular biology through pathology, radiology, surgery, radiotherapy, medical oncology, paediatric oncology to epidemiology. We welcome original articles, reviews, case reports and letters, and we would be pleased to publish abstracts from meetings held to encourage multidisciplinary research.

The publication of *Sarcoma* coincides with the first steps towards truly international collaboration in this field. Professor Suit and his colleagues on both sides of the Atlantic have set up the Connective Tissue Oncology Society, whose aims are also to encourage international collaboration and the exchange of ideas and data. That is the only way in which to make progress in this heterogeneous group of diseases. Without cooperation the important questions such as the role of adjuvant chemotherapy in high-grade sarcoma cannot be answered. Progress in the management of sarcomas depends on relevant high-quality science being available to all interested workers: *Sarcoma* is here to provide a reliable focus for this work.

Some 1500 relevant articles are published each year. We would like to encourage you to consider submitting yours to *Sarcoma.* We hope that you will enjoy its content and, by working together with us, drive forward improvements in the diagnosis and management of this diverse group of diseases.

Lastly, I would like to thank all members of the Editorial Board who have helped me with turning the idea of a dedicated journal into reality.

Correspondence to: M. Robinson, Editor, *Sarcoma*, YCRC Department of Clinical Oncology, Weston Park Hospital NHS Trust, Whitham Road, Sheffield S10 2SJ, UK. Tel: +44 114 226 5221; Fax: +44 114 226 5511; E-mail: m.h.robinson@sheffield.ac.uk.

1357-714X/97/010003-01 ©1997 Journals Oxford Ltd

